# Outcome predictors in patients with temporal lobe epilepsy after temporal resective surgery

**DOI:** 10.1186/s42494-024-00190-3

**Published:** 2024-12-09

**Authors:** Jiabin Yu, Yinchao Li, Xuan Xie, Liming Cheng, Shaofang Zhu, Lisen Sui, Youliang Wu, Xuemin Xie, Haitao Xie, Xiaojing Zhang, Chun Chen, Yingying Liu

**Affiliations:** 1 Department of Epilepsy Center, The Second Affiliated Hospital, Guangzhou University of Chinese Medicine, Guangzhou, 510120 China; 2 Department of Neurology, The Seventh Affiliated Hospital, Sun Yat-sen University, Shenzhen, 517108 Guangdong Province China; 3 Guangzhou University of Chinese Medicine, Guangzhou, 510006 China; 4 Brain Disease Function Department, The Second Affiliated Hospital, Guangzhou University of Chinese Medicine, Guangzhou, 510120 China; 5 Department of Neurology, Third Affiliated Hospital, Sun Yat-sen University, 600 Tianhe Road, Guangzhou, 510630 Guangdong China

**Keywords:** Temporal lobe epilepsy, Resective surgery, Predictors, Prognosis

## Abstract

**Background:**

Temporal lobe epilepsy is one of the most common types of partial epilepsy. Although surgical treatment has led to significant improvements in seizure-free rates, nearly one-third of patients still have poor seizure control after surgery. Moreover, the long-term outcome is less favorable compared to short-term outcome, with 48–58% of patients experiencing seizures five years after surgery. The aim of this study was to investigate the surgical outcomes and the predictive value of prognostic factors associated with poor surgical outcomes in temporal lobe epilepsy patients receiving surgery.

**Methods:**

We retrospectively reviewed 94 patients undergoing temporal resective surgery in the Epilepsy Center of Guangdong Provincial Hospital of Traditional Chinese Medicine between July 2016 and July 2020. Patient information including age, gender, personal and family history, as well as preoperative and postoperative clinical data (clinical type and duration of disease) was collected.

**Results:**

The differences of postoperative clinical efficacy in both seizure free group and non-seizure free group patients were observed. A log-rank test was used for univariate analysis, and a Cox proportional hazard model was used for multivariate analysis. Ninety-four patients were followed up for at least 1 years. At 12 months of follow-up, 71 (75.5%) patients achieved Engel class I, 5 (5.3%) patients were classified as Engel class II, 5 (5.3%) patients were classified as Engel class III, and 13 (13.8%) patients were classified as Engel class IV. Univariate analysis and multivariate Cox regression analysis indicated that the postoperative EEG abnormalities were significantly correlated with seizure recurrence and were significant independent predictive factors, with a hazard ratio of 12.940.

**Conclusions:**

The relapse rate in our study was similar to commonly reported overall rates in temporal lobe epilepsy patients receiving surgery. Anterior temporal lobectomy is a reliable treatment option for temporal lobe epilepsy patients. Postoperative electroencephalograph abnormalities are independent risk factors for poor surgical prognosis.

## Background

 Temporal lobe epilepsy (TLE) is one of the most common types of partial epilepsy, in which the epileptogenic foci are generally located in the temporal lobe. TLE accounts for 60% of refractory epilepsy cases and surgical resection is considered an effective treatment for medically refractory epilepsy [1]. Although about 5–42% of TLE patients have response to conventional therapeutic interventions, up to 50–70% of patients are resistant to anti-seizure medications [2–5]. Anterior temporal lobectomy can effectively reduce the seizure recurrence rate in epilepsy patients, with 60–70% of drug-refractory TLE patients achieving seizure freedom at 1–2 years post-resection, and almost 50% of patients experiencing prolonged seizure freedom up to 10 years [6–8]. In a retrospective study of resective surgery in medial TLE with hippocampal sclerosis (MTLE/HS), 80.3% and 81.4% of older and younger patients achieved Engel I outcome at the final follow-up, with no significant difference between the two groups. The postoperative cognitive and psychiatric results were comparable between the two groups. The rates of major complications were also comparable (3.3% in the older group and 2.7% in the younger group). This study provided grade 3 evidence that resective surgery for MTLE/HS patients aged 50 years or older is as effective and safe as it is for younger patients. Therefore, it should be regarded as the primary treatment choice for drug-resistant cases [9]. A multicenter randomized clinical trial for patients with drug-resistant TLE showed that patients with drug-resistant TLE who received early surgical treatment had significantly better prognosis than those who continued to receive drug treatment. Among patients with drug-resistant MTLE/HS, the seizure-free rate after epilepsy surgery can reach 70% [10]. Despite significant improvements in seizure-free rates with surgical treatment, almost one-third of patients with TLE still have poor seizure control after surgery, and the long-term outcome is less favorable than the recent outcome, with 48–58% of patients still experiencing seizures 5 years after surgery [11, 12]. The use of imaging and other examination methods have improved the therapeutic effects of surgery in patients with epilepsy. Many factors are considered to be associated with the effectiveness of surgical treatment in patients with TLE, including age of onset, temporal courses, as well as electroencephalograph (EEG), imaging, pathological and serological characteristics [13–17]. However, some domestic and international studies have confirmed that the age of onset, temporal courses, seizure frequency and age at surgery are not closely related to the prognosis of patients [18–24]. Patients with an earlier age of epilepsy onset are more than three times likely to achieve a good surgical prognosis than other patients [25]. However, some researchers have proposed that the age of onset actually reflects the prognostic value of hippocampal sclerosis due to the following reasons [26]. First, most patients with an earlier age of onset have typical characteristics of hippocampal sclerosis, including unilateral hippocampal atrophy shown on magnetic resonance imaging (MRI) [27] and focal seizure onset shown in a electroencephalogram [28, 29]. Univariate analysis of data from multiple studies suggest that patients with a longer history of epilepsy may have a poorer surgical prognosis [30]. The relationships of clinical factors such as the frequency of epileptic seizures, the history of febrile convulsions, and the presence or absence of aura, with surgical prognosis, remain illusive. Therefore, reliable predictors of patient outcome after surgery are needed to provide a reliable theoretical basis for the screening of patients for surgery.

In this study, we retrospectively reviewed post-operative outcomes of TLE patients who underwent resective surgery at our epilepsy center from July 2016 to July 2020, and investigated factors associated with the prognosis of surgery in patients with TLE.

## Methods

### Study subjects

This study was approved by the Human Ethics Committee of Guangdong Provincial Hospital of Traditional Chinese Medicine, and written informed consent was obtained from each participant.

The inclusion criteria were as follows: ① Epilepsy cases confirmed according to the International League Against Epilepsy criteria based on clinical description of seizures, post-ictal physical examination when available, and (video) EEG; ② Patients who did not show efficient control of seizures with correct use of at least one anti-seizure medication at adequate doses; ③ Presurgical evaluation was made with non-invasive procedures only; and ④ Patients were diagnosed with focal cortical dysplasia (FCD) (including neocortical lesions and medial temporal lobe lesions) with/without hippocampal sclerosis (i.e., FCD type I, FCD type II, FCD type IIIa) by post-operative pathological examination.

The exclusion criteria were as follows: ① Patients with postoperative pathological diagnosis of pathological types other than FCD type I, II, and IIIa; ② Patients with invasive assessment (subdural electrodes, Stereoelectroencephalography, etc.) before resective surgery; ③ Patients who had received palliative surgery such as vagus nerve stimulation; and ④ Patients were not followed up to one year after surgery.

### General information

Ninety-four patients with TLE who underwent surgery at our epilepsy center from July 2016 to July 2020 were included in this study. Patient information was recorded on case-record forms by an epilepsy specialist, including gender, age, age of onset, duration, whether being early onset of generalized tonic-clonic seizures, seizure frequency, previous history, imaging findings (including cranial MRI and positron emission tomography [PET]), preoperative EEG, whether the seizure initiation site was the site of resection, whether the intraoperative discharge range was completed within the resection range, and postoperative pathology. All patients were followed up every two months until the end of the study period. Patients were required to return for follow-up appointments.

### Preoperative assessment

Patients received comprehensive presurgical evaluations consisting of a complete neurologic examination, ictal and inter-ictal EEG, ^18^F-FDG PET/CT, and brain MRI (3.0 T) during the first admission period. The surgical approach and the extent of resection were recommended by consultation among neurologists, neurosurgeons, neuroradiologists and electrophysiologists.

### Operational method

Anterior temporal lobectomy with amygdalohippocampectomy was performed in 94 patients. Operations were performed under general anesthesia with endotracheal intubation. Fifteen minutes prior to cortical EEG monitoring, the anesthesiologist reduced the dosage of the anesthetic to lower the depth of anesthesia to facilitate intraoperative EEG monitoring. After the EEG monitoring was completed, the anesthesia depth was deepened again. After confirming the extent of discharge and the number and type of discharges, we determined the posterior border of temporal lobectomy, first separated the lateral fissure, and opened the temporal horn inward from the middle temporal gyrus at the intended posterior border of the resection. Anatomical landmarks such as lateral ventricular lateral sulcus, lateral parabrachial bulge, choroid plexus, hippocampal dispersion, choroidal fissure, and terminal stripe were identified under the microscope, and the anterior temporal lobe was resected along the lateral ventral sulcus to fully expose the hippocampal structures. The hippocampal head was resected approximately 3 cm posteriorly, and amygdala, uncus of temporal lobe and medial structures of temporal lobe were removed. The cortical EEG was rechecked, and the epileptiform discharges largely disappeared.

### Postoperative pathology

The resected tissues were sent to the Pathology Department of our hospital for pathological examination (hematoxylin-eosin staining and immunohistochemical examination).

### Follow up and clinical efficacy evaluation

A total of 94 patients were discharged and followed up while receiving therapeutic dosages of the same anti-seizure medications as given pre-operatively. Patients returned to the hospital every 6 months for long-term video-EEG (VEEG) monitoring (≥ 16 h), and all were followed up for more than 1 year. If patients remained seizure-free for 1 years and showed no epileptiform discharges on EEG after surgery, anti-seizure medications were gradually discontinued. All patients were followed up regularly for a period of approximately 1 year after surgery. Surgical outcomes were categorized into the following grades according to the International League Against Epilepsy classification: Grade I, no seizures or only aura seizures; Grade II, very few seizures (≤ 2 episodes/year); Grade III, significant improvement with ≥ 75% reduction of seizures; and Grade IV, no significant improvement with < 75% reduction of seizures.

### Statistical methods

Statistical analysis was performed using the Statistical Package for the Social Sciences version 25.0 software. Enumeration data are expressed in percentage, and measurement data are expressed as mean ± standard deviation. Survival analysis was performed to determine seizure recurrence. Survival time was defined as the period from surgery to seizure recurrence or to the end of the study. The censored value was defined as the first seizure after surgery. A survival curve was constructed to describe the recurrence rate after surgery. A log-rank test was used for univariate analysis, and a Cox proportional hazard model was used for multivariate analysis to identify the risk factors for seizure recurrence. Parametric data were analyzed with one-way analysis of variance (ANOVA) and Bonferroni tests. Non-parametric data were analyzed with the Kruskal-Wallis method and the Mann-Whitney U test. All statistical tests were two-tailed. *P* < 0.05 was considered as statistically significant.

## Results

### General clinical information

Among the 94 patients, 50 (53.2%) were males and 44 (46.8%) were females. The onset age was 14.94 ± 10.14 years (1–55.3 years), the age at surgical treatment was 26.06 ± 10.48 years (4–56 years), the duration of disease was 11.12 ± 8.56 months (0.7–45.0 months), and the mean monthly seizure frequency was 11.11 ± 23.00 (0.3–200 seizures/month). There were 56 patients with a history of hypoxia at birth, 69 patients with hippocampal sclerosis, and 91 patients receiving PET examination. All patients had received standard anterior temporal lobectomy (Table 1).

**Table 1:** Characteristics of patients with temporal lobe epilepsy receiving temporal resective surgery

Characteristics	Number of patients (%)/Mean ± SD (range)
Total number	94
Male	50 (53.2%)
Age at surgery, years	26.06 ± 10.48 (4–56)
Age at seizure onset, years	14.94 ± 10.14 (1–55.3)
≤ 18	67 (71.3%)
>18	27 (28.7%)
Disease duration, months	11.12 ± 8.56 (0.7–45.0)
Side of surgery	
Left	48 (51.1%)
Right	46 (48.9%)
Early appearance of GTCS	57 (60.6%)
No	37 (39.4%)
Yes	57 (60.6%)
Seizure frequency, monthly	
< 10 times	71 (75.5%)
≥ 10 times	23 (24.5%)
Preoperative intermittent EEG abnormality	
Unilateral	71 (75.5%)
Bilateral	23 (24.5%)
Typical symptomatology	
Negative	20 (21.3%)
Positive	74 (78.7%)
Consistency of resection site with ictal EEG initiation region	
No	20 (21.3%)
Yes	73 (77.77%)
Unkown	1 (1.1%)
Low-metabolic-activity regions in PET/CT	
Multiple	69 (73.4%)
Single	22 (23.4%)
PET/CT not performed	3 (3.2%)
Intraoperative discharge range being within the resection range	
No	17 (18.1%)
Yes	77 (81.9%)
Drug-resistant epilepsy	
No	37 (39.4%)
Yes	57 (60.6%)
Postoperative EEG abnormalities	39 (41.5%)
Lesion location	
Mesial temporal lobe	61 (64.9%)
Neocortical temporal lobe	33 (35.1%)

*PET* Positron emission tomography, *GTCS *Generalised tonic-clonic seizures, *EEG *Electroencephalography, *MRI* Magnetic resonance

### Postoperative pathological results

All resected specimens were subjected to pathological examination. Two cases had pathological type of FCD I, including 1 case of FCD Ib and 1 case of FCD Ic; 1 case had pathological type of FCD IIa, and 90 cases had pathological type of FCD III with most of them being FCD IIIa.

### Follow-up results

All 94 patients were regularly followed-up. A progressive decrease in seizure-freedom frequency were observed over time. According to the Engel scale, 75.5% (71/94) of the 94 patients achieved seizure freedom (Engel I), 5.3% (5/94) achieved Grade II, 5.3% (5/94) in Grade III and 13.8% (13/94) in Grade IV (Table 2).

**Table 2:** Characteristics of epilepsy patients who experienced seizure freedom after temporal resective surgery

	Seizure free group, *n* (%)
Patients	70 (100%)
Male	35 (56.1%)
Adult onset	18 (21.2%)
Early appearance of GTCS	37 (52.9%)
Disease duration before surgery < 6 months	64 (91.4%)
History of hypoxia	47 (67.1%)
MRI-positive	69 (98.6%)
Multiple brain regions showing slow metabolic activity in PET/CT	9 (12.9%)
Hippocampal sclerosis	56 (80%)
Typical Symptomatology	63 (90%)
Preoperative bilateral intermittent EEG abnormality	11 (15.7%)
Postoperative EEG abnormality	39 (41.5%)
Resection site consistent with ictal EEG initiation regions	59 (84.3%)
Intraoperative discharge range being within the resection range	64 (91.4%)
Lesion location in mesial temporal lobe	17 (24.3%)
Drug*-*resistant epilepsy	37 (52.9%)

*PET *Positron emission tomography, *GTCS *Generalised tonic-clonic seizures, *EEG *Electroencephalography, *MRI *Magnetic resonance imaging

### Predictors of seizure outcomes

Univariate analysis was performed to determine the risk factors for seizure recurrence.

Early appearance of generalised tonic-clonic seizures (GTCS), history of hypoxia, MRI-positive, slow metabolic activity in multiple brain regions in PET/CT, hippocampal sclerosis, typical symptomatology, preoperative bilateral intermittent EEG abnormality, postoperative EEG abnormality, resection site consistent with ictal EEG initiation regions, intraoperative discharge range within the resection range, lesion location in the mesial temporal lobe, and drug-resistant epilepsy were identified as risk factors of seizure recurrence (Table 3). For multivariate analysis, the variables with a *P* value < 0.1 in univariate analysis were subjected to the Cox regression analysis. The results showed that postoperative EEG abnormality was an independent predictor of prognostic outcomes in patients receiving TLE surgery (Table 4).

**Table 3:** Univariate analysis by log rank test of risk factors for seizure recurrence

Variables	Patients *n*	Relapsed *n*	*P* value
Male	50	15	0.363
Adult onset	27	9	0.223
Early appearance of GTCS	57	20	0.011
Age at surgery < 18 years	67	15	0.223
Monthly seizure frequency < 10 times	71	19	0.735
Disease duration before surgery < 20 months	83	19	0.117
History of hypoxia	56	9	0.006
MRI-positive finding	85	16	< 0.001
Slow metabolic activity in multiple brain regions in PET/CT	22	13	< 0.001
Hippocampal sclerosis	69	13	0.014
Typical symptomatology	74	11	< 0.001
Preoperative bilateral intermittent EEG abnormality	23	12	< 0.001
Postoperative EEG abnormality	39	22	< 0.001
Resection site consistent with ictal EEG initiation regions	73	14	0.008
Intraoperative discharge range within the resection range	77	13	< 0.001
Lesion location being mesial temporal lobe	61	8	< 0.001
Drug-resistant epilepsy	57	20	0.011

*PET* Positron emission tomography, *GTCS *Generalised tonic-clonic seizures, *EEG *Electroencephalography, *MRI *Magnetic resonance imaging

**Table 4:** Multivariate analysis of risk factors for seizure recurrence using the Cox proportional hazard model

	HR	95% CI for HR		*P* value
		Lower	Upper	
Early appearance of GTCS	2.367	0.477	11.737	0.292
History of hypoxia	0.746	0.263	2.115	0.581
MRI-positive finding	1.225	0.303	4.959	0.776
Slow metabolic activity in multiple brain regions in PET/CT	2.004	0.824	4.876	0.125
Hippocampal sclerosis	0.515	0.16	1.653	0.265
Typical symptomatology	0.650	0.116	3.642	0.624
Preoperative bilateral intermittent EEG abnormality	1.290	0.407	4.091	0.665
Postoperative EEG abnormality	12.940	2.705	61.915	**< 0.001**
Resection site consistent with ictal EEG initiation regions	0.871	0.252	3.017	0.828
Intraoperative discharge range within the resection range	0.872	0.268	2.838	0.82
Lesion location in the mesial temporal lobe	0.998	0.167	5.969	0.998
Drug-resistant epilepsy	2.492	0.682	9.102	0.167

*HR* Hazard ratios, *PET* Positron emission tomography, *GTCS *Generalised tonic-clonic seizures, *EEG *Electroencephalography, *MRI* Magnetic resonance imaging

## Discussion

TLE, with epileptiform discharges originating in the temporal lobe including the hippocampus, amygdala, parahippocampal gyrus, and lateral temporal neocortex, is the most common type of epilepsy and accounts for more than 50% of adult epilepsy [31–35]. In contrast, more than half of the surgically ineffective cases suffered epilepsy recurrence within 6 months after surgery, and more than 95% of patients suffered epilepsy recurrence within 2–5 years [36–38]. Therefore, the seizure-freedom rate decreased more rapidly within 5 years after surgery, and remained relatively stable beyond 5 years [39–41]. Early postoperative recurrence may be due to incomplete removal of the epileptogenic zone due to inaccurate localization.

### Presurgical evaluations and surgical outcomes

Positive MRI findings allow better localization of the epileptogenic zone, leading to a better prognosis for surgery [42]. The univariate analysis results of this study indicated that positive MRI findings were correlated with surgical prognosis. The objective of epilepsy surgery is to completely remove the epileptogenic zone from where the seizures originate, thereby controlling epileptic seizures. The main aim of presurgical evaluation is to precisely locate the epileptogenic zone in patients with medically intractable epilepsy. In this study, PET-CT was conducted on 92 patients. In practical scenarios, PET is highly advantageous in locating the epileptogenic focus, particularly when MRI results are negative or when there is inconsistency between MRI and VEEG results. Our univariate analysis results demonstrated that hypo-metabolic changes in a single brain region in PET served as a protective factor for prognosis. Generally, the epileptogenic zone in MRI-negative epilepsy is diffuse and extensive, making it challenging to localize and determine the extent of resection, even with the help of intracranial electrode EEG. The rate of seizure control after epilepsy surgery is closely associated with the extent of resection of the epileptogenic focus, and the completeness of resection has been proven to be one of the most crucial factors in the formation of seizures. Previous literature has reported a postoperative seizure-freedom rate of over 70% in patients with epilepsy associated with lesions and less than 50% in those without associated lesions, which is completely in line with the results of this study [43]. Preoperative assessment of the epileptogenic focus localization is targeted and has a guiding role. The extent of the lesion is also the most basic and core scope of surgical resection, which facilitates the planning of the resection area. This study suggested that whether the seizure-phase EEG onset area is consistent with the resection site and whether the intraoperative discharge range is completely within the resection range have an important influence over the outcome, and both factors facilitate successful determination of the epileptic zone in patients. Tatum et al. performed 4 preoperative assessments in 39 patients with medial TLE, including ictal EEG temporal rhythmicity origin, PET/SPECT and Wada test, and found that only the ictal EEG temporal rhythmicity origin was associated with a good prognosis for surgery [44]. Lau et al. compared patients with negative cranial MRI finding and those with sclerosis on one side of the hippocampus, and found that good postoperative seizure control was associated with θ origination on one side of the EEG during seizures, but not with the presence of hippocampal sclerosis. When MRI showed negative findings, ictal EEG value is a good predictive tool for prognosis [44–46]. Cranial MRI and ictal EEG examination are two important objective assessment approaches for preoperative evaluation. Our study implied that the preoperative evaluation should focus on cranial MRI and seizure EEG for the prognosis of patients. The principal objective of epilepsy surgery is to completely remove the area where the seizures originate, thereby achieving full control of the seizures. Hence, the precise localization of the epileptogenic zone is the main aim of preoperative evaluation for epilepsy. Currently, localization of the epileptogenic zone is primarily accomplished through the electro-clinical-anatomical theory. The preoperative evaluation process is divided into two stages: a noninvasive evaluation and an invasive evaluation, which needs to be conducted based on the noninvasive evaluation. The two significant noninvasive tests are cranial MRI and scalp long-range VEEG, which must be completed in all epilepsy patients considered for surgery. To facilitate the detection of hippocampal sclerosis, cortical dysplasia, and other potential epileptogenic lesions, cranial MRI must encompass sequences such as T1-weighted images, T2-weighted images, T2-FLAIR images, and coronal hippocampal thinning scans. Additionally, cranial MRI enhancement is necessary when tumor lesions are suspected. In scalp long-range video EEG monitoring, the interictal and ictal EEG epileptiform discharges are analyzed to determine the origin of epileptic discharges, and the captured ictal symptomatology is analyzed to establish the hypothesis of the network structure of seizure generation and propagation, forming a flawless electro-clinical-anatomical hypothesis to precisely identify the epileptogenic zone, enabling direct surgical intervention. When the non-invasive findings are inconsistent, a perfect electro-clinical-anatomical hypothesis cannot be established; subsequently, further refinement by invasive intracranial EEG is required to further delineate the epileptogenic zone.

### Clinical features and prognostic factors

The influence of clinical factors such as age of onset, age at surgery, seizure frequency, and disease duration on the prognosis of surgery is still controversial. Most of the literature stated that age at onset, age at surgery, frequency of seizures, and duration of disease have no effect on prognosis, which is the same as the results of this study [47]. Some studies have indicated that the age at onset actually reflects the prognostic value of hippocampal sclerosis. This is because the majority of patients with an earlier age at onset possess typical characteristics of hippocampal sclerosis, which is a factor contributing to a favorable surgical prognosis [48]. Only a few studies have suggested that the earlier the age of surgery, the better the postoperative prognosis for patients [49]. Univariate analysis of several earlier studies suggests a poorer prognosis for surgery in patients with a long disease history [17]. However, multifactorial analysis in some similar studies showed no correlation between disease duration and prognosis [50]. Factors influencing the outcome of surgical treatment for drug-resistant TLE are complex and diverse. This study discovered that typical symptomatology, no early manifestation of GTCS, and non-drug-resistant epilepsy were predictors of a favorable prognosis. In patients with drug-refractory TLE who experienced postoperative recurrence, the likelihood of aura-only seizures secondary to GTCS was lower, and the long-term outcome was superior to that in patients with CPS/GTCS as the predominant type of recurrent epilepsy [51].

### Prognosis and pathology

The types of epilepsy surgical pathology include hippocampal sclerosis, FCD, low-grade tumors, vascular malformations, ischemic lesions, or inflammation. Researchers generally agreed that non-specific pathological findings are associated with a relatively poor prognosis for surgery. In a longitudinal study of 371 anterior temporal lobectomy patients, the seizure-freedom rate after surgery was approximately 44% in patients with non-specific pathological findings and 64% in those with specific pathology at a mean follow-up of 8 years [52]. However, the relationship between the type of specific pathology and surgical prognosis is unclear. Earlier studies suggested a good prognosis for surgery in hippocampal sclerosis, but many recent studies pointed to no difference in postoperative seizure-freedom rates between hippocampal sclerosis and other specific pathological types [13, 52].

In this study, we compared the prognostic impact of the presence or absence of hippocampal sclerosis findings, and found a statistically significant difference in the postoperative seizure-freedom rate of 75.5% for those with positive pathology and 38.9% for those without positive pathology, which was not consistent with the results of the bulk study. This discrepancy may be explained by the small sample size of this study, which needs to be further expanded for analysis.

### Limitations

There were some limitations in this study. First, the study was a retrospective analysis and the inherent bias associated with the retrospective nature of this study could not be avoided. Second, the sample size was relatively small, and a larger sample is needed to draw more definitive conclusions. Third, the patients’ quality of life and neuropsychological outcomes were not assessed. Despite these limitations, the present study provided important supporting information for the surgical treatment of TLE patients undergoing resection.

## Conclusions

A total of 94 patients with drug-resistant or focal-related epilepsy receiving surgery were included in this study, with a postoperative follow-up of approximately 1 year. The long-term postoperative seizure-freedom rate was 75.5%. Our study revealed that postoperative EEG abnormalities were closely related to the prognosis of surgery. The combination of MRI, VEEG, intraoperative cortical EEG and/or intracranial electrode EEG should be used to precisely locate the epileptogenic focus, thereby improving the detection rate and facilitate complete resection of the epileptogenic focus, which can improve the prognosis of patients and their quality of life.

## Data Availability

All the supporting data related to this work are available from the corresponding author on reasonable request.

## Abbreviations

EEG: Electroencephalograph
FCD: Focal cortical dysplasia
GTCS: Generalised tonic-clonic seizures
MRI: Magnetic resonance imaging
MTLE/HS: Mesial temporal lobe epilepsy with hippocampal sclerosis
PET: Positron emission tomography
TLE: Temporal lobe epilepsy
